# COVID-19 pandemic and the consumption of self-care products for pediculosis capitis in Portugal: an interrupted time-series analysis

**DOI:** 10.1007/s00436-024-08258-2

**Published:** 2024-06-08

**Authors:** Diogo Almeida, Antonio Teixeira Rodrigues, Jose Pedro Guerreiro, Ewa Bałkowiec-Iskra, Robert Vander Stichele, Bruno Sepodes, Carla Torre

**Affiliations:** 1 Faculdade de Farmácia, Universidade de Lisboa, Avenida Professor Gama Pinto, 1649-003 Lisbon, Portugal; 2 Centre for Health Evaluation & Research (CEFAR-INFOSAUDE), National Association of Pharmacies (ANF), Lisbon, Portugal; 3 Life and Health Sciences Research Institute (ICVS), School of Medicine, University of Minho, Campus de Gualtar, Braga, Portugal; 4 Department of Experimental and Clinical Pharmacology, Medical University of Warsaw, Warsaw, Poland; 5 Unit of Clinical Pharmacology, Faculty of Medicine, Ghent University, Ghent, Belgium; 6 Laboratory of Systems Integration Pharmacology, Clinical and Regulatory Science, Research Institute for Medicines of the University of Lisbon (iMed.ULisboa), Lisbon, Portugal

**Keywords:** Pediculosis capitis, COVID-19, Time series analysis, Real-world data

## Abstract

This study aims to assess the effect of the COVID-19 pandemic on the consumption of self-care products for pediculosis capitis management, in Portugal. A segmented regression analysis of interrupted time series (March 2020) was performed from January 2017 to August 2023 to analyze the short- and long-term impact of the COVID-19 pandemic on the consumption of pediculicides and related products. Monthly rates of absolute consumption were estimated by community pharmacies’ dispensing records. Portuguese municipalities were organized into quintiles according to their purchasing power index and percentage of youth, to study the association of these social and demographic variables on the sale of these products. COVID-19 pandemic significantly reduced the sales of products indicated for pediculosis. Since the start of the pandemic, an absolute decrease of 21.0 thousand packages was observed in the monthly average consumption (*p* < 0.0001) compared to the pre-pandemic period. After this reduction, the average monthly trend increased in the pandemic period in comparison with the previous period, although not significant (267.0 packages per month, *p* = 0.1102). Regions with higher disposable income and more young people were associated with higher sales of these products. The outbreak of the COVID-19 pandemic has had a notable impact on the sales of self-care products for pediculosis capitis in Portugal, in the short term. The lockdowns and other isolation measures implemented to control the spread of the virus may have led to a decrease in the number of head lice cases, consequently resulting in a reduction in sales of products.

**Supplementary Information:**

The online version contains supplementary material available at 10.1007/s00436-024-08258-2.

## Introduction

Epidemiological data on pediculosis is limited in Portugal. Nonetheless, a meta-analytic study emphasized that the prevalence of this condition was estimated in 19% among school-age students. The studies included in this analysis indicated possible risk factors, such as parents’ literacy, family size, financial condition, hair length, and hygiene measures (Hatam-Nahavandi et al. 2020). Despite the importance of evaluating and monitoring the clinical scenario and symptoms of pediculosis capitis (e.g., scalp pruritus and irritation, the most common symptoms), many patients claim that the most concerning issues are emotional consequences and psychological stress (Devore et al. 2015). Thus, this impact in both psychological and social domains should also be closely followed to avoid consequences, such as anxiety and the feeling of isolation. These issues are even more concerning in children because they can deteriorate their development and impact their ability to socialize with peers (Leung et al. 2022).

Transmission of pediculosis capitis is highly dependent on direct contact with an infested individual (Mumcuoglu et al. 2009). During the COVID-19 pandemic, several restrictions and other public health strategies were implemented, which have affected the dynamics of certain clinical conditions, such as infectious diseases (Chauhan 2020). The Portuguese government declared a state of emergency on March 18th, 2020, and with this declaration, concrete public health measures were implemented. The obligation of social distancing, lockdown, limitation of the mass movement of people, and the use of masks were some examples of the measures applied (Pais and Taveira 2020). After these efforts and considering the great adherence of the Portuguese population to vaccination, the government declared the end of the state of emergency on September 30th, 2022 (Governo de Portugal 2022).

COVID-19 influence on self-manageable conditions has not yet undergone thorough analysis, particularly regarding infestations caused by head lice. Moreover, some evidence points to an increase in resistance to topical treatments in recent years (Leung et al. 2022). Hence, this study aims to assess the effect of the COVID-19 pandemic on the pattern of self-care products consumption for pediculosis capitis management in Portugal and to analyze how sales from pharmacies located in regions with different purchasing power indexes and percentages of youth are associated with these demographic and social variables.

## Methods

### Study design

A segmented regression analysis of interrupted time series (March 2020) was performed from January 2017 to August 2023 to analyze the short- and long-term impact of the COVID-19 pandemic on the consumption of self-care products for pediculosis capitis. Monthly rates of absolute consumption were estimated by community pharmacies’ dispensing records. Portuguese municipalities were organized into quintiles according to both their purchasing power index and percentage of youth (people under 20 years old) to explore the influence of these variables on the dispensing of self-care products for pediculosis capitis. Both social variables were extracted according to the data available from the Portuguese National Statistics Institution (Instituto Nacional de Estatística 2021a, 2021b).

### Setting and data sources

The Portuguese community pharmacies’ dispensing records were obtained through the hmR Information System and analyzed by CEFAR (Centre for Health Evaluation and Research). This representative national database compiles health products-dispensing data (prescription and non-prescription) from approximately 2460 (84%) out of 2920 community pharmacies in Portugal (Domingues et al. 2023).

A list containing 254 self-care products for pediculosis capitis was extracted from the mentioned database and each product was verified in terms of its suitability to manage pediculosis capitis. This procedure was carried out considering the information made available by the producer on product labeling, package leaflets, or in official communication channels of the company. This list comprises a wide variety of products, such as over-the-counter medicines, medical devices, and cosmetics. In terms of presentation, these products were classified as balm, wax, shampoo, conditioner, cream, cream-liquid, hair tie, cutaneous foam, hair band, gel, lotion, mask, mousse, oil, comb, electric comb, solution, cutaneous solution, spray, lotion-spray, and kits containing 2 or 3 of the products described. Table 1 presents the distribution of the products analyzed in different categories, according to the categories of the pharmacies’ database used. In Supplement I, an extensive table illustrates the list of details for each product included in the analysis.

**Table 1:** Distribution of the products studied in terms of presentation, market, and treatment category

Classification categories	Quantity of products, *n* (%) Total = 254
Presentation	
Shampoo	62 (24.4%)
Lotion	48 (18.9%)
Kit	38 (15.0%)
Spray	34 (13.4%)
Comb	18 (7.1%)
Cutaneous solution	15 (5.9%)
Other*	39 (15.3%)
Market	
Medical device	110 (43.3%)
Cosmetics and hygiene	88 (34.7%)
Biocide	21 (8.3%)
Over-the-counter medicine	10 (3.9%)
Other	25 (9.8%)
Treatment category	
Topical	183 (72.0%)
Mechanical	36 (14.2%)
Topical and mechanical	35 (13.8%)

*Other presentations include balm, conditioner, cream, cream-liquid, cutaneous foam, electric comb, gel, hair band, hair tie, mask, mousse, oil, spray-lotion and wax

### Outcome measures

The number of package sales of self-care products indicated for pediculosis capitis was the main outcome measure. Additionally, the volume of sales was then studied to assess its possible association with social and demographic variables of different Portuguese municipalities (purchasing power index and percentage of youth).

### Data analysis

A population-based, single arm, uncontrolled, interrupted time series analysis was conducted to assess longitudinal effects of the COVID-19 pandemic. This analysis considers previous trends in the outcome and studies its modification in response to the intervention studied (Wagner et al. 2002). Despite being recommended to use a control group that is not submitted to the same intervention, a single group interrupted time series assures its internal validity by considering the pre-intervention segment as the control of the post-intervention segment (Soumerai et al. 1993). Additionally, it is recommended to consider at least 24 monthly data points to avoid correlation errors, since one data point can be more similar to the one measured one year ago, than the ones measured in other months of the same year (Wagner et al. 2002).

The application of segmented regression analysis to the interrupted time series data allows to statistically infer how much an intervention altered the outcome studied in the short and long term (Wagner et al. 2002). In this study, the “intervention” corresponded to the beginning of the COVID-19 pandemic in Portugal (March 2020, when the Portuguese government declared a state of emergency). Therefore, the 80 months period was divided in two segments: 38 months before March 2020 and 42 months subsequently (from April 2020 to August 2023). A graph was then plotted to visualize the trends in these two periods studied and monthly sales were expressed in volume (number of packages sold). A regression model of the interrupted time series analysis was applied (Linden 2015). Autoregressive integrated moving average models were used to control for seasonality and autocorrelation (Wagner et al. 2002).

Afterwards, the data were analyzed in quintiles to study how sales from pharmacies located in regions with different percentages of youth and purchasing power index were associated with these demographic and social variables. The Portuguese municipalities were organized in two groups of quintiles: one group of municipalities regarding the percentage of youth and a second regarding the purchasing power index. The first quintiles correspond to the municipalities with the lowest percentage of youth and purchasing power index, while the fifths aggregate data from the municipalities with more young people and purchasing power index, respectively. The volume of sales and the average volume of sales per pharmacy were then studied for both groups of quintiles.

The analysis was conducted using SAS Enterprise Guide v7.15 (SAS Institute, Cary, NC). The statistical significance adopted was *α* = 0.05.

## Results

Results show that prior to the COVID-19 pandemic (from January 2017 until March 2020), a positive but not significant trend was observed in the average monthly sales of these products. Since the start of the pandemic period (i.e., March 2020), an absolute decrease of 21.0 thousand packages was observed in the monthly average consumption of these products (*p* < 0.0001), in comparison to the pre-pandemic period. After this reduction, the average monthly trend increased in the pandemic period in comparison with the previous period, although not significantly (267.0 packages per month; *p* = 0.1102). Nonetheless, the maximum reached in the pandemic period remained much lower when compared to the pre-pandemic values.

**Fig. 1:**
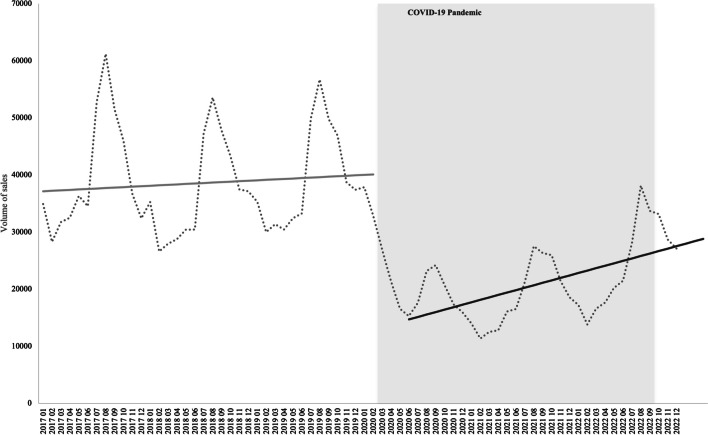
Volume of sales of products for pediculosis capitis, 2017–2023

A clear seasonality in the sales data was observed, with peaks occurring, generally, in August. A sharp decline in sales can be depicted when the pandemic was declared by the national government (absolute decrease of 21.0 thousand packages; *p* < 0.0001) (Fig. 1).

**Fig. 2:**
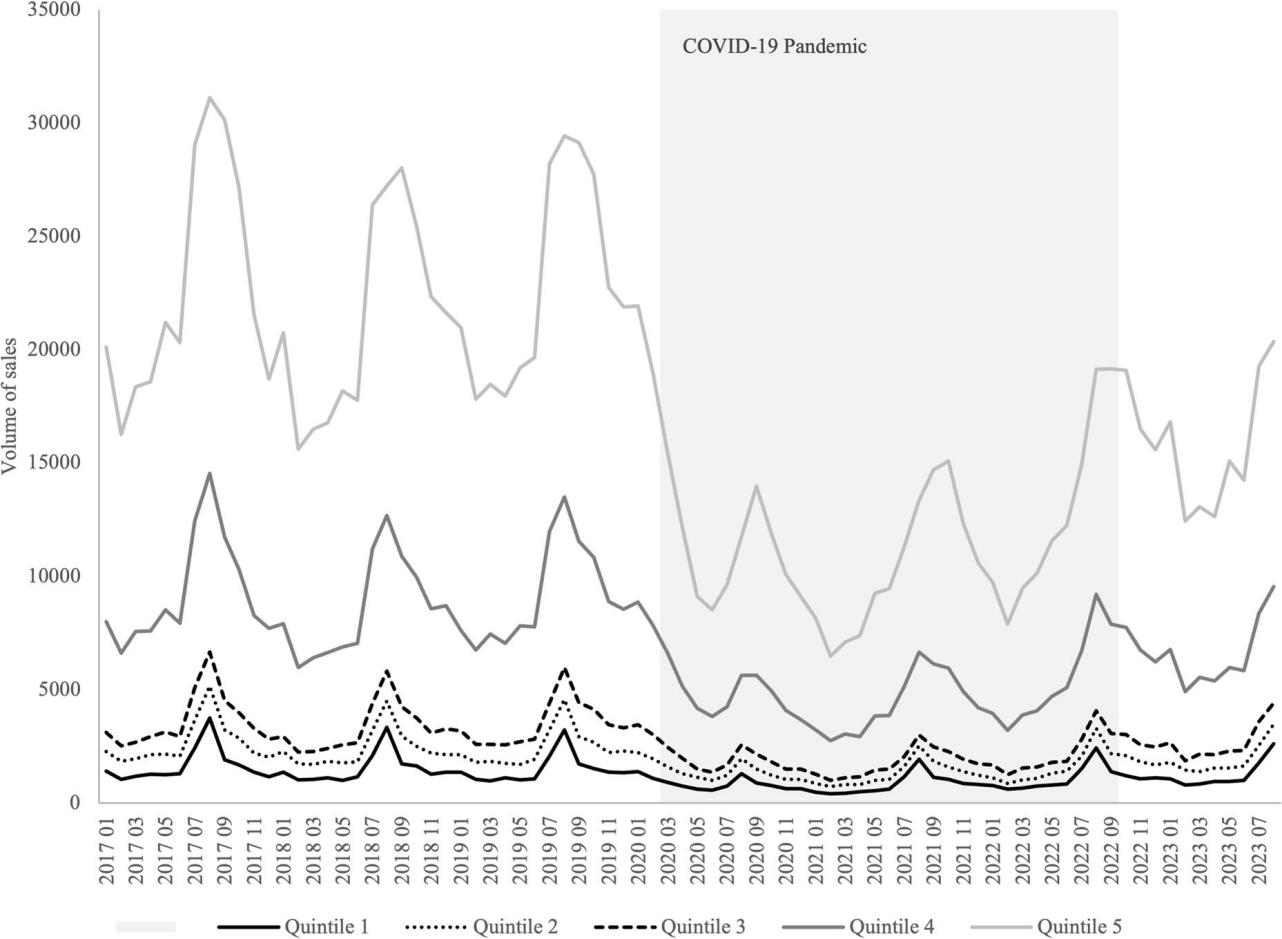
Quintiles of municipalities on the purchasing power index

In terms of the social and demographic, similar trends regarding the total volume of sales were observed. Figure 2 illustrates the quintiles regarding the purchase power index. Results showed that the 1st quintile (lowest purchase power index) presented the lowest volumes of sales, while the 5th (highest purchase power index) has the largest. This trend was maintained during the pandemic period.

A significant decrease in sales can be observed for every quintile (*p* < 0.0001) as a consequence of the beginning of the COVID-19 pandemic in March 2020. Considering the long-term effect of the pandemic, the measurements were only significant for the lowest quintiles (quintiles 1 and 2) where sales increased by 19.0 (*p* < 0.0034) and 24.8 (*p* < 0.0116) thousand packages, respectively (Table 2).

**Table 2:** Short- and long-term effects of the pandemic on volume of sales distributed in quintiles of municipalities according to the respective purchasing power index

Quintile**	Short-term effect	*p*-value	Long-term effect	*p*-value
1	− 839.4	< 0.0001*	19.0	0.0034*
2	− 1320.0	< 0.0001*	24.8	0.0116*
3	− 1955.0	< 0.0001*	25.9	0.0877
4	− 4841.0	< 0.0001*	54.7	0.1824
5	− 11,905.0	< 0.0001*	136.9	0.1656

*Significant *p*-value

**The higher the quintile, the more municipalities with a higher purchasing power index it contains

Regarding the percentage of youth, municipalities with less young people (quintile 1) present an inferior total volume of products sold, whereas the municipalities with a higher percentage of youth (quintile 5) appear to have the highest numbers of volume. This trend maintained stable during the pandemic period.

## Discussion

Sales of self-care items for managing pediculosis capitis in the Portuguese community pharmacy setting decreased substantially as a result of the measures adopted to combat the COVID-19 pandemic, indicating that the pandemic affected the dynamics of head lice infestations.

This decrease in sales might suggest a reduction in the incidence of pediculosis capitis during the pandemic, corroborating the findings of other authors. A descriptive study showed that the results of a questionnaire distributed to the population of Buenos Aires indicated a significantly decreased prevalence of pediculosis in children. The authors suggested that this finding might be associated with mandatory public health measures, such as lockdowns, which prevented children to contact each other in the school environment (Galassi et al. 2021). Additionally, a preliminary study on the evolution of the number of sales of pediculicide units in the largest pharmacy chain in Israel displayed a decrease in the trend coinciding with the start of the pandemic. Authors also attributed this event to the measures implemented by the government to control the spread of the pandemic (Mumcuoglu et al. 2022). A study conducted in France investigated the impact of public health measures on the sale of medicines, both prescription and over-the-counter. The study analyzed dispensing data from 60% of all French pharmacies and found that the implementation of the measures led to a decrease in the number of products sold, particularly during the first lockdown (Launay et al. 2022).

Considering the outcomes described in the studies mentioned, a similar pattern might have happened in Portugal. Pandemic restrictions limited social interaction which is essential for the transmission of head lice (Mumcuoglu et al. 2009; Pais and Taveira 2020). Thus, a decrease in consumption of these products was observed (absolute decrease of 21.0 thousand packages; *p* < 0.0001).

In the pandemic, the average monthly trend increased compared with the previous period, although not significantly (267.0 packages per month, *p* = 0.1102) and the increasing slope of the average monthly sales observed after the start of the pandemic, suggest an increasement in sales possibly due to a higher social interaction, as the restrictions, especially lockdowns, were progressively ending (Komori et al. 2022).

Furthermore, our study results suggest that the volume of sales is impacted by the municipalities’ purchasing power index. Financial hardship strongly influences the initiation of treatment and its adherence, making economically better placed people less hesitant to buy health products to better manage their health (Schommer et al. 2003). A Brazilian cross-sectional study illustrates these results. Out of the participants included in the research who reported having difficulties in accessing medication, 45.6% stated that lack of financial resources corresponds to the main reason (Tiguman et al. 2020). In Portugal, over-the-counter products for pediculosis capitis have varying prices and are not reimbursed by the government. Thus, these products may not be easily accessible for some families, especially in regions with a lower purchasing power index.

The short-term effect of the pandemic, translated in a significant reduction of sales, can be perceived for every quintile. Restrictions were implemented at a national level, impacting all regions independently of their economic power. After the start of the pandemic, municipalities with a lower purchasing power index (quintiles 1 and 2) were the only ones which experienced a significant increase in sales of products for the infestation. Other social and demographic variables of this specific population might have contributed to the rise in pediculosis capitis cases and, consequently, in sales, designating this topic as a focal point for future investigation.

Regarding the percentage of people aged under 20 years on sales, municipalities with more young people appear to be the ones in which pharmacies generate more sales of products for head lice infestation. This event was expected and can be explained by pediculosis being more prevalent among children (Fu et al. 2022; Mumcuoglu et al. 2009). Head-to-head contact consists of the main route of pediculosis capitis transmission. Thus, the school setting corresponds to an important source of transmission since children behavior make them more susceptible of getting in contact with infested heads (Mumcuoglu et al. 2009).

### Strengths and limitations

Firstly, the database used pharmacy dispensing data (prescription and non-prescription health products) that assure representative national coverage (84% of the country’s pharmacies). Other published studies generally used smaller databases with limited national extrapolation. Thus, we can assume that the results obtained are much closer to the reality of the country. Additionally, the time series analysis comprised a period of more than 3 years after the beginning of the pandemic. Subsequently, the study was able to incorporate data points from the entire pandemic period (from beginning to end). This fact was particularly relevant thus allowing to interpret the results with the COVID-19 trends completed.

Some limitations deserved to be mentioned. The study’s ability to comprehend the COVID-19 pandemic’s long-term effects is limited by the lack of data points from the post-pandemic period. Therefore, future analyses may be needed when enough time passes after the pandemic to collect more data points. The products studied are also sold in other places, such as parapharmacies, which were not taken into account in this study. However, considering the nature and indications of the products concerned, it is expected that in these other retailers the trends were similar. Finally, it is impossible to exclude the biases implied by people’s consumption attitudes from the analysis, due to the nature of the data used (i.e., pharmacy dispensing records), and also by the gap in knowledge regarding individuals’ behavior in their homes.

## Conclusions

The COVID-19 pandemic and its mitigation measures affected largely people’s routines and consequently the dynamics of certain diseases. Pediculosis requires direct contact for the parasite to be transmitted and this study demonstrated that the COVID-19 pandemic public health measures caused a significant reduction in sales of products indicated for pediculosis in Portugal, suggesting a decrease in pediculosis capitis cases.

Regions with more disposable income were associated with higher sales of the products studied since possessing more financial resources might have facilitated the access to health products. Despite not affecting the general sales trend between quintiles (the municipalities with a higher purchasing power index continued to be those in which pharmacies generated more sales of products for pediculosis), the COVID-19 pandemic caused a significant decrease in all regions, regardless of their economic condition.

### Supplementary Information

Below is the link to the electronic supplementary material.

Supplementary file1 (DOCX 43 KB)

## Data Availability

Purchasing power index and percentage of youth data were retrieved from the Statistics Portugal public available database. Products indicated for pediculosis data used in the study were provided by hmR Information System®. Data will be shared at reasonable request to the corresponding author and under hmR Information System® agreement.

## Abbreviations

CEFAR: Centre for Health Evaluation and Research
COVID-19: Coronavirus disease 2019
