# Supplementary material for: COVID-19 pandemic and the consumption of self-care products for pediculosis capitis in Portugal: an interrupted time-series analysis
Source: Parasitol Res. 2024 Jun 8;123(6):235. doi: 10.1007/s00436-024-08258-2 (PMC11162394; doi:10.1007/s00436-024-08258-2)
Supplement: Supplementary file 1 — Supplementary file1 (DOCX 43 KB) [file 436_2024_8258_MOESM1_ESM.docx]

**SUPPLEMENT I**

**Table 1 –** List of products included in the analysis.

| **Market** | **Brand** | **Product Name** | **Presentation** | **Treatment Category** |
| --- | --- | --- | --- | --- |
| CH | Acofar | Acofarderm Ch Ol Arvore Cha 200ml | Shampoo | T |
| CH | Advancis | Advancis P Zero Ch Pos Trat Piolhos100 Ml | Shampoo | T |
| CH | Advancis | Advancis P Zero Ch Prot Piolh/Lend 100ml | Shampoo | T |
| MD | Advancis | Advancis P Zero Loc Piolhos100+Pente x2 | Kit | TM |
| MD | Advancis | Advancis P Zero Loc Spray Piolh100ml+Pent | Kit | TM |
| MD | Advancis | Advancis P Zero Locao Piolhos 100ml+Pente | Kit | TM |
| O | Assy | Assy Pente Piolhos Lendeas | Comb | M |
| CH | Aya | Aya Ch Espuma Suave 150 Ml | Shampoo | T |
| CH | Aya | Aya Kit Champô + Pente Piolhos/Lêndeas | Kit | TM |
| MD | Aya | Aya Pente Piolhos Lendeas | Comb | M |
| O | Biostop | Biostop Pente Elect Piolhos | Electric comb | M |
| B | Charlieu | Charlieu Sh Anti Poux 125 Ml | Shampoo | T |
| O | Cutilfar | Cutilfar Pente Piolho Duralon | Comb | M |
| CH | Desintan P | Desintan P Cr Liq 60ml | Cream | T |
| MD | Dexal | Dexal 100ml +Pente | Cutaneous solution | TM |
| O | Dimor | Dimor Pente Piolhos Com Lupa | Comb | M |
| MD | Ducray | Ducray Itax Locao Piolhos 75ml | Lotion | T |
| CH | Ducray | Ducray Itax Spray Desembaracad 100 Ml | Spray | T |
| CH | Ducray | Ducray Piolhos Itax Sh Suave 150 Ml | Shampoo | T |
| CH | Ducray | Ducray Piolhos Kit Anti Piolhos 08 | Kit | TM |
| CH | Ducray | Ducray Piolhos Kit Itax Anti Piolhos | Kit | TM |
| CH | Ecokid | Ecokid Locao Locao Cab Com Piolhos | Lotion | T |
| MD | Elimax | Elimax Ch Piolhos/Lend 100ml | Shampoo | T |
| MD | Elimax | Elimax Ch Piolhos/Lend 250ml | Shampoo | T |
| CH | Elimax | Elimax Ch Prevent Piolhos 200Ml | Shampoo | T |
| CH | Elimax | Elimax Locao Piolhos/Lend 100ml | Lotion | T |
| MD | Elimax | Elimax Pure Power Lc Piolho/Lend 100 | Lotion | T |
| O | Estipharm | Estipharm Pente Metalico Antipiolhos | Comb | M |
| MD | Farline | Farline Pente Anti Piolhos Lendeas 3X1 | Comb | M |
| O | Floris | Floris Pente Elect Piolhos Lend | Electric comb | M |
| CH | FullMarks | Fullmarks Ch Pos Trata Piolhos 150ml | Shampoo | T |
| MD | FullMarks | FullMarks Duo Loção Piolhos/Lêndeas 2 x 100 ml | Lotion | T |
| MD | FullMarks | Fullmarks Loc Piolh/Lend100Mldu0+50%Des | Lotion | T |
| CH | FullMarks | Fullmarks Locao +Ch Pos Trata Desc 50% | Lotion | T |
| MD | FullMarks | Fullmarks Locao Piolh/Lendeas 100ml | Lotion | T |
| CH | FullMarks | FullMarks Loção Piolhos/Lêndeas + Champô Pós tratamento Piolhos | Kit | T |
| CH | FullMarks | Fullmarks Spray 150+Ch150Ml+Desc50% | Spray | T |
| MD | FullMarks | Fullmarks Spray 150Ml+of Ch 150ml | Spray | T |
| MD | FullMarks | Fullmarks Spray Anti-Piolhos/Lendeas150ml | Spray | T |
| CH | Gotitas de Oro | Gotitas Oro Ch Piolhos 500ml | Shampoo | T |
| CH | Gotitas de Oro | Gotitas Oro Locao Piolhos 250ml | Lotion | T |
| CH | Hedrin | Hedrin Ch Tudo Em 1 200Ml | Shampoo | T |
| CH | Hedrin | Hedrin Detecao Remocao Lendea100 | Kit | TM |
| CH | Hedrin | Hedrin Once Spray Gel 100Ml | Spray | T |
| CH | Hedrin | Hedrin Pente Detecao Piolhos | Comb | M |
| CH | Hedrin | Hedrin Protect Go Spray 120Ml | Spray | T |
| CH | Helan | Helan Occhio Pid 37l Locao Prev Piolh 100m | Lotion | T |
| CH | Helan | Helan Occhio Pid 37o Ol Piolh Spray 100ml | Oil | T |
| CH | Helan | Helan Occhio Pid 37sh Ch Esp Piolho 200ml | Shampoo | T |
| MD | Holon | Holstop Locao Piolhos 100Ml | Lotion | T |
| CH | Insigny | Insigny Kids Ch Piolhos 200ml | Shampoo | T |
| CH | Insigny | Insigny Kids Lc Desembarac Piolh 200ml | Lotion | T |
| MD | Lauroderme | Lauroderme Nitolic Prev Plus Spray75 | Spray | T |
| MD | Lauroderme | Lauroderme Nitolic Sist Trat Piolhos | Kit | TM |
| CH | Lenpiox | Lenpiox Ch Prevent 300ml | Shampoo | T |
| CH | Lenpiox | Lenpiox Loção piolhos 100ml+Pente | Kit | TM |
| CH | Liceguard | Liceguard Ch Removed Piolhos 120ml | Shampoo | T |
| MD | Liceguard | Liceguard Spray Repel Piolhos 30ml | Spray | T |
| MD | Licener | Licener Ch Piolhos Lendeas 100ml | Shampoo | T |
| MD | Licinin | Licinin Locao Piolhos Lend 100ml | Lotion | T |
| MD | Lipuk | Lipuk Locao Cap Piolhos 150 Ml | Lotion | T |
| MD | Lipuk | Lipuk Locao Piolho+Pente 150 Ml | Kit | TM |
| O | Loon | Loon Fita Elastic Cab Piolhos Bolinha | Hair band | M |
| O | Loon | Loon Fita Elastic Cab Piolhos Bolinha X2 | Hair band | M |
| O | Loon | Loon Fita Elastic Cab Piolhos Coracao | Hair band | M |
| O | Loon | Loon Fita Elastic Cab Piolhos Coracao X2 | Hair band | M |
| O | Loon | Loon Fita Elastic Cab Piolhos Estrela | Hair band | M |
| O | Loon | Loon Fita Elastic Cab Piolhos Estrela X2 | Hair band | M |
| O | Loon | Loon Fita Elastic Cab Piolhos Lisa X2 | Hair band | M |
| O | Loon | Loon Fita Elastic Cab Piolhos Preta | Hair band | M |
| MD | Magnien | Magnien Elastic Cab Piolhos X2 | Hair tie | M |
| O | Magnien | Magnien Pente Anti Piolhos | Comb | M |
| MD | Magopid | Magopid Ch Activo 200 Ml | Shampoo | T |
| MD | Magopid | Magopid Locao Shock 125 Ml | Lotion | T |
| O | Maniquick | Maniquick Pente Anti Ftiriase | Comb | M |
| B | Musside | Musside Sh 50 G | Shampoo | T |
| MD | Neemosan | Neemosan Locao Cap Piolhos 125 Ml | Lotion | T |
| MD | Quitoso | Neo Quitoso Plus Sol 50ml Leve2 Pague1 | Cutaneous solution | T |
| MD | Quitoso | Neo Quitoso Plus Sol Cut 100Ml+Desc 50% | Cutaneous solution | T |
| MD | Quitoso | Neo Quitoso Plus Sol Cut Piolhos Lend 50ml | Cutaneous solution | T |
| MD | Quitoso | Neo Quitoso Plus Sol Cut Piolhos Lend100ml | Cutaneous solution | T |
| MD | Quitoso | Neo Quitoso Plus Sol Cut+Of Toalhasx2+Fita | Kit | T |
| MD | Quitoso | Neo Quitoso Plus100+Toalha+Fita+Desc50% | Kit | T |
| MD | Quitoso | Neo Quitoso Sol Cut Piolhos Lend 50ml | Cutaneous solution | T |
| CH | Neoparaderma | Neoparaderma Ch Espuma 100 Ml | Shampoo | T |
| MD | Neoparaderma | Neoparaderma Mousse 100 Ml | Shampoo | T |
| MD | Newell | Newell Locao Antipiolhos 100Ml | Lotion | T |
| CH | Newell | Newell Protect Ch Arvore Cha Org 250Ml | Shampoo | T |
| CH | Newell | Newell SpraY Protect Maca 250Ml | Spray | T |
| CH | Newell | Newell Spray Protect Morango 250Ml | Spray | T |
| OTC | Nix | Nix, 10 mg/g-60 mL x 1 creme frasco | Cream | T |
| CH | No-Lice | No-Lice Elasticos Prot Cab X4 | Hair tie | M |
| CH | Nosa | Nosa Attack Locao Vinagre Quass 150ml | Lotion | T |
| CH | Nosa | Nosa Protect Cera Pentear Arvore Cha 100ml | Wax | T |
| CH | Nosa | Nosa Protect Ch Maca Arvore Cha 250ml | Shampoo | T |
| CH | Nosa | Nosa Protect Ch Morang Arvore Cha 250ml | Shampoo | T |
| CH | Nosa | Nosa Protect Ch Toranja Arvore Cha 250ml | Shampoo | T |
| CH | Nosa | Nosa Protect Masc Arvore Cha Morang100 | Mask | T |
| CH | Nosa | Nosa Protect Masc Morang Arvor Cha250Ml | Mask | T |
| CH | Nosa | Nosa Protect Ol Arvore Cha 100% 16ml | Oil | T |
| CH | Nosa | Nosa Protect Sp Maca Arvore Cha 250ml | Spray | T |
| CH | Nosa | Nosa Protect Sp Morang Arvore Cha 250ml | Spray | T |
| CH | Nosa | Nosa Protect Sp Pesseg Arvore Cha 250Ml | Spray | T |
| CH | Nosa | Nosa Protect Sp Textil Arvore Cha 250ml | Spray | T |
| CH | Nosa | Nosa Protect Sp Toranja Arvore Cha 250ml | Spray | T |
| MD | Oficinal | Oficinal Duo Champô 2 x 100 ml | Shampoo | T |
| MD | Oficinal | Oficinal Limov Ch Piolhos 100Ml | Shampoo | T |
| MD | Oficinal | Oficinal Limov Ch Piolhos 100Ml Duo 50% | Shampoo | T |
| B | Olicide | Olicide Locao 50 Ml | Lotion | T |
| B | P Zero | P Zero Locao Parasita 60 Ml | Lotion | T |
| B | Para Derma | Paraderma Sh Banho 120 Ml | Shampoo | T |
| MD | Paramitex | Paramitex Locao 100 Ml | Lotion | T |
| MD | Paramitex | Paramitex Locao 50 Ml | Lotion | T |
| MD | Paramitex | Paramitex Pack Locao 50ml X 2 | Lotion | T |
| CH | Paranix | Paranix Ch Condic 100ml | Shampoo | T |
| MD | Paranix | Paranix Ch Piolhos200+Of Gel Localiz | Shampoo | T |
| CH | Paranix | Paranix Ch Pos Tratam Piolh 100ml | Shampoo | T |
| MD | Paranix | Paranix Ch Prot Piolho/Lend 200ml | Shampoo | T |
| MD | Paranix | Paranix Ch Trat200Ml+Ch Prot200Ml | Shampoo | T |
| B | Paranix | Paranix Ch Tratam 200+SY Amb225+Desc15E | Kit | T |
| MD | Paranix | Paranix Ch Tratam Piolhos 200ml | Shampoo | T |
| MD | Paranix | Paranix Champô Tratam Piolhos/Lêndeas + Pente 200 ml com Oferta de Caixa Lápis Cor | Kit | TM |
| MD | Paranix | Paranix Champô Tratamento + Champô Protector 200 ml com Desconto de 50% | Shampoo | T |
| MD | Paranix | Paranix Champô Tratamento com Oferta de Spray Repelente | Shampoo | T |
| MD | Paranix | Paranix Extr Fort Ch Trat 200+Of Sp Rep | Shampoo | T |
| MD | Paranix | Paranix Extr Fort Ch Trat 200Ml+Of Ch P | Shampoo | T |
| MD | Paranix | Paranix Extra Forte Ch Tratamento 200Ml | Shampoo | T |
| MD | Paranix | Paranix Extra Forte Lc Tratamento 100Ml | Lotion | T |
| MD | Paranix | Paranix Extra Forte Sp Tratamento 100Ml | Spray | T |
| MD | Paranix | Paranix Gel Localiz Lend 150ml | Gel | T |
| MD | Paranix | Paranix Gel Localiz Lend150Ml+25%Desc | Gel | T |
| MD | Paranix | Paranix Lc Piolhos/Lend Desc 2E | Lotion | T |
| MD | Paranix | Paranix Loção Piolhos/Lêndeas 100 ml + Pente | Lotion | TM |
| MD | Paranix | Paranix Loção Sensitive Piolhos/Lêndeas com Oferta de Repelente | Kit | T |
| MD | Paranix | Paranix Mousse Piolhos/Lend 100ml | Mousse | T |
| MD | Paranix | Paranix Pack Spray+Pent+ Repel | Kit | TM |
| CH | Paranix | Paranix Repel Spray 100 Ml | Spray | T |
| CH | Paranix | Paranix Repel Spray 100Ml Desc20% | Spray | T |
| MD | Paranix | Paranix Sensitive Locao Piolhos/Lend 150 Ml | Lotion | T |
| MD | Paranix | Paranix Spray + Pente com Oferta de Champô Pós-Tratamento | Kit | TM |
| MD | Paranix | Paranix Spray + Pente com Oferta de Spray Repelente | Kit | TM |
| MD | Paranix | Paranix Spray C/Pente Piolh 100Ml | Spray | TM |
| MD | Paranix | Paranix Spray C/Pente100+Of Ch Prot200 | Kit | TM |
| MD | Paranix | Paranix Spray Piolhos 60 ml + Pente | Kit | TM |
| CH | Paranix | Paranix Spray Repelente 100 ml com  Desconto de 2€ | Spray | T |
| MD | Paranix | Paranix Spray S/Pente Piolh 100ml | Spray | T |
| MD | Paranix | Paranix Spray sem Pente com Oferta de Champô Pós-tratamento | Spray | T |
| CH | Paranix | Paranix Spray sem Pente com Oferta de Repelente | Spray | T |
| MD | Pára Pio | Parapio Duo Lp Locao 150 Ml | Lotion | T |
| MD | Pára Pio | Parapio Flash Locao Gel 120ml | Lotion | T |
| B | Pára Pio | Parapio Locao 100 Ml | Lotion | T |
| CH | Pára Pio | Parapio Protect Ch Po Lendeas Piolhos 30g | Shampoo | T |
| B | Pára Pio | Parapio Sh 100 Ml | Shampoo | T |
| B | Pára Pio | Parapio Vap 100 G | Spray | T |
| CH | Pararoma | Pararoma Champoo Sh 100 Ml | Shampoo | T |
| CH | Pararoma | Pararoma Locao Locao 30 Ml | Lotion | T |
| CH | Parasidose | Parasidose Balsamo Amaciador 150ml | Balm | T |
| MD | Parasidose | Parasidose Cuid Tratamento 100ml | Lotion | T |
| MD | Parasidose | Parasidose Cuid Tratamento 200Ml | Cutaneous solution | T |
| MD | Parasidose | Parasidose Locao Piolhos 50 Ml | Lotion | T |
| CH | Parasidose | Parasidose Pack Duo Bálsamo Amaciador | Balm | T |
| MD | Parasidose | Parasidose Pente Piolhos | Comb | M |
| CH | Parasidose | Parasidose Sh Desembaracador Lendose | Shampoo | T |
| B | Parasidose | Parasidose Sh Piolhos 200 Ml | Shampoo | T |
| OTC | Parasidose | Parasidose, 2 mg/g-200 mL x 1 champô frasco | Shampoo | T |
| MD | Parasitack | Parasitack LC Spray 100ML+Pente Metal | Spray | TM |
| CH | Parasitack | Parasitack Prevent Ch 100Ml | Shampoo | T |
| B | Pasita | Pasita Locao 60 Ml | Lotion | T |
| OTC | Piky | Piky (100mL), 38,33 mg/mL x 1 sol cut | Cutaneous solution | T |
| OTC | Piky | Piky (100mL), 38,33 mg/mL x 1 sol cut | Cutaneous solution | T |
| OTC | Piky | Piky (200mL), 38,33 mg/mL x 1 sol cut | Cutaneous solution | T |
| OTC | Piky | Piky (50mL), 38,33 mg/mL x 1 sol cut | Cutaneous solution | T |
| OTC | Piky | Piky, 38,33 mg/mL x 100 sol cut | Cutaneous solution | T |
| OTC | Piky | Piky, 38,33 mg/mL x 200 sol cut | Cutaneous solution | T |
| OTC | Piky | Piky, 38,33 mg/mL x 60 sol cut | Cutaneous solution | T |
| MD | Puressentiel | Puressentiel Locao Anti-Piolhos 100ml | Lotion | T |
| MD | Puressentiel | Puressentiel Locao Piolhos Pente 100Ml | Kit | TM |
| MD | Puressentiel | Puressentiel Piolhos Ch Masc 150+Pente | Kit | TM |
| CH | Puressentiel | Puressentiel Pouxdoux Ch Fq Desc50%2ªUn | Shampoo | T |
| CH | Puressentiel | Puressentiel Pouxdoux Ch Uso Freq200ml | Shampoo | T |
| CH | Puressentiel | Puressentiel Sos Spray Repel Piolh75ml | Spray | T |
| MD | Puressentiel | Puressentiel Spray Piolhos 100ml | Spray | T |
| OTC | Quitoso | Quitoso , 10 mg/g Recipiente pressurizado  100 g Esp cutan | Cutaneous foam | T |
| CH | Quitoso | Quitoso Care Ch 150 Ml | Shampoo | T |
| MD | Quitoso | Quitoso Care Pente Metali Tripla Accao | Comb | M |
| B | Quitoso | Quitoso Cr Liq 50 Ml | Cream-liquid | T |
| CH | Quitoso | Quitoso Locao 60 Ml | Lotion | T |
| B | Quitoso | Quitoso Locao Locao Parasita 60 Ml | Lotion | T |
| B | Quitoso | Quitoso Locao Locao Parasita Neb 75 Ml | Lotion | T |
| MD | Quitoso | Quitoso Neo pack solução cutânea + Oferta Pente | Kit | TM |
| MD | Quitoso | Quitoso Neo pack solução cutânea + Pente + Champô | Kit | TM |
| MD | Quitoso | Quitoso Neo solução cutânea Piolhos/Lêndeas 50 ml com Desconto de 20% com Oferta de Toalha | Cutaneous solution | T |
| CH | Quitoso | Quitoso Sh 60 Ml | Shampoo | T |
| O | Robi Comb | Robi Comb Pente Elect Piolhos | Electric comb | M |
| O | San Up | San Up Pente Piolhos Lendeas | Comb | M |
| B | Sempiol | Sempiol Locao 75 Ml | Lotion | T |
| B | Seta | Seta Ch 150 Ml | Shampoo | T |
| B | Seta | Seta Locao 75 Ml | Lotion | T |
| CH | Seta Natural | Seta Natural Ch Freq Prot Piolho 250ml | Shampoo | T |
| CH | Seta Natural | Seta Natural Kit Champô 125 ml + Loção 125 ml + Pente piolhos | Kit | TM |
| CH | Seta Natural | Seta Natural Locao Piolhos 125 Ml | Lotion | T |
| CH | Seta Natural | Seta Natural Sh Piolhos 125 Ml | Shampoo | T |
| MD | Sinlice | Sinlice Fr Piolhos Lendeas 100 Ml | Cream | T |
| CH | Stop | Stop Champo 125ml | Shampoo | T |
| CH | Arkopharma | Stop Kids Prevent Oleo Arvore Cha 20ml | Oil | T |
| CH | Arkopharma | Stop Kids Prevent Oleo Arvore Do Cha 15ml | Oil | T |
| CH | Stop Piolhos | Stop Piolhos Champo 125ml | Shampoo | T |
| MD | Stop Piolhos | Stop Piolhos Gel Cab Curto 100ml+Pente | Kit | TM |
| MD | Stop Piolhos | Stop Piolhos Gel Cabelos Curtos 100ml | Gel | T |
| MD | Arkopharma | Stop Piolhos Kit Completo Cab Comprido | Lotion | T |
| MD | Stop Piolhos | Stop Piolhos Kit Completo Cab Curto | Kit | TM |
| MD | Stop Piolhos | Stop Piolhos Loc Cab Long 100ml+Pente | Kit | TM |
| MD | Stop Piolhos | Stop Piolhos Loção C/Pente+Repel Dsc25% | Kit | TM |
| MD | Stop Piolhos | Stop Piolhos Locao Cabelos Longos100ml | Lotion | T |
| MD | Stop Piolhos | Stop Piolhos Locao Cap 100ml+Pente | Kit | TM |
| MD | Stop Piolhos | Stop Piolhos Locao Piolho 100ml | Lotion | T |
| MD | Stop Piolhos | Stop Piolhos Locao+Oft Ch+Pente | Lotion | TM |
| MD | Arkopharma | Stop Piolhos Pack Familiar Desc 50% 2U | Lotion | T |
| CH | Stop Piolhos | Stop Piolhos Pack Piolh Elimin Previne | Kit | TM |
| B | Stop Piolhos | Stop Piolhos Spray Repulsivo 125ml | Spray-lotion | T |
| MD | Stop Piolhos | Stop PiOlhs Natu Locao100Ml C/ Of Pente | Kit | TM |
| CH | Stop Repel | Stop Repel Sp Cabelo 100Ml+Of Ch 125Ml | Spray | T |
| CH | Stop Repel | Stop Repel Spray Cabelo 100Ml | Spray | T |
| CH | Stop Repel | Stop Repel Spray Cabelo 30Ml | Spray | T |
| CH | Th Pharma | Th Pharma Petit Prev2-Phase Cond Sp 300ml | Conditioner | T |
| CH | Th Pharma | Th Pharma Petit Prevent Ch Escolar 300ml | Shampoo | T |
| CH | Tiox | Tiox Ch Prev 250 Ml | Shampoo | T |
| MD | Tiox | Tiox Pente Piolhos Lendeas | Comb | M |
| O | Titania | Titania Pente Piolhos 1819 PHB | Comb | M |
| MD | Tricovel | Tricovel Neopidok Oleo 75Ml+Ch 150Ml | Kit | T |
| MD | Tricovel | Tricovel Neopidok Oleo Piolhos 75ml | Oil | T |
| CH | Tricovel | Tricovel Pidoko Ch 150 Ml | Shampoo | T |
| MD | YapaPou | Yapapou Locao Spray Piolhos 100ml | Spray | T |
| MD | Zap | Zap X Elastico Cab Anti-Piolhos X2 | Hair tie | M |
| MD | Zap | Zap X Pente Eletronico Piolhos | Comb | M |
| MD | Zap | Zap X Pente Manual Antipiolhos | Comb | M |
| B | Zas | Zas Sh Parasitario 100 Ml | Shampoo | T |
| O | Ztop | Ztop Fitas Cab Piolhos Br | Hair band | M |
| O | Ztop | Ztop Fitas Cab Piolhos Pr | Hair band | M |
| O | Ztop | Ztop Fitas Cab Piolhos Rosa | Hair band | M |
| B | Ztop | Ztop Nopic Spray Repel Piolhos 200ml | Spray | T |
| O | Ztop | Ztop Pente Elect Piolhos | Electric comb | M |
| CH | Advancis | Advancis P-Zero Protect Spray 120ml | Spray | T |
| CH | Advancis | Advancis P-Zero Protect Ch 120ml | Shampoo | T |
| B | Paranix | Paranix Spray Ambiente 225Ml | Spray | T |
| CH | Newell | Newell Antipiolhos Kit 2Em1 Ch120/Lc100 | Kit | T |
| MD | Arkopharma | Stop Piolhos Cuidad Total Lc+Sp Repelen | Kit | T |
| O | Acofarma | Nesira Pente antipiolhos Premium +0M | Comb | M |
| MD | Piolhout | Piolhout Touca Anti-Piolhos X2+Pente | Kit | TM |
| MD | Pára Pio | ParaPio Pro Loção Lêndeas Piolhos 100Ml | Lotion | T |
| O | Arkopharma | Stop Pente com Lupa | Comb | M |
| MD | Stop Piolhos | Stop Piolhos Ch Tratamento Pente 200ml | Shampoo | TM |
| MD | Arkopharma | Stop Piolhos Pack Trata&Cuida Loc100+Ch | Kit | T |
| MD | kidsner | Kidsner Lc Piolhos Bela 4A+ 75Ml | Lotion | T |
| B, Biocide; CH, Cosmetics and hygiene; MD, Medical device; O, Other; OTC, Over-the-counter medication; T, Topical; M, Mechanical; TM, Topical and mechanical. | | | | |
